# Analysis of CAT Gene Family and Functional Identification of OsCAT3 in Rice

**DOI:** 10.3390/genes14010138

**Published:** 2023-01-04

**Authors:** Wenxiang Jiang, Qing Ye, Zheng Wu, Qiuyun Zhang, Lianhong Wang, Jialin Liu, Xiafei Hu, Dandan Guo, Xiaoqing Wang, Zelin Zhang, Haohua He, Lifang Hu

**Affiliations:** 1 College of Agriculture, Jiangxi Agricultural University, Nanchang 330045, China; 2 Key Laboratory of Crop Physiology, Ecology and Genetic Breeding, Ministry of Education, Jiangxi Agricultural University, Nanchang 330045, China

**Keywords:** rice, catalase, gene function, stress

## Abstract

Catalase (CAT) is an important antioxidant enzyme in plants that plays a key role in plant growth and stress responses. CAT is usually encoded by a small gene family that has been cloned and functionally studied in some species, such as *Arabidopsis*, wheat and cucumber, but its specific roles in rice are not clear at present. In this study, we identified three CAT family genes (*OsCAT1*, *OsCAT2* and *OsCAT3*) in the rice genome and performed a systematic bioinformatics analysis. RT−PCR analysis revealed that *OsCAT1–OsCAT3* was primarily expressed in vegetative tissues such as roots, stems and leaves. Since *OsCAT3* showed the highest expression level among the three *OsCAT* genes, we then focused on its related functions. OsCAT3 prokaryotic expression protein has an obvious ability to remove H_2_O_2_. The *OsCAT3_crispr_* plant was short and had a low survival rate, the leaves were small with brown lesions, and the activities of the CAT, POD and SOD enzymes were significantly reduced. A microarray analysis showed that differentially expressed genes were primarily enriched in toxin metabolism and photosynthesis. This study laid a foundation for further understanding the function of the rice *OsCAT* gene.

## 1. Introduction

Catalase (CAT) is one of the key enzymes in the biological defense system during biological evolution [1]. It can effectively scavenge hydrogen peroxide produced during plant photorespiration and mitochondrial electron transfer by catalyzing the transfer of electrons and breaking down hydrogen peroxide into water and oxygen and plays an important role in plant defense and stress responses, delaying plant ageing and controlling the redox balance in plant cells [2,3,4]. The relative molecular weight of CAT generally ranges from 200 to 340 kD and is usually composed of four subunits [5]. Each subunit contains a haem molecule and a peroxisomal targeting signal (PTS1) peptide sequence at the carboxyl terminus to recognize the peroxisome [6,7]. According to the difference in the CAT catalytic center structure, CAT can be divided into the iron porphyrin structure CAT (FeCAT) and the manganese porphyrin structure CAT (MnCAT) [8].

Plant catalase is usually encoded by a small gene family; tobacco, *Arabidopsis*, maize, pumpkin, cucumber and barley were each found to contain three members of this family, and two members each were identified in cottonseed and *Hordeum vulgare* [9,10,11,12,13,14].

A series of studies have shown that different CAT family members exhibited distinct spatiotemporal expression patterns, as well as different responses to developmental and environmental stimuli. In tobacco, CAT1 and CAT2 were specifically expressed in non-senescent leaves, whereas CAT3 was detected in both non-senescent and senescent leaves [15].

Maize *ZmCAT1* and *ZmCAT3* were expressed throughout the whole kernel process, but *ZmCAT2* was only expressed during the later stage of kernel development [16]. The transcription level of CAT1–CAT3 in pepper is related to the circadian rhythm and different stresses [17]. *AtCAT1* and *AtCAT2* are primarily expressed in leaves and fruit pods, while *AtCAT3* is expressed in stems and roots [18]. *AtCAT2* is induced by drought and cold stress, whereas *AtCAT3* is induced by ABA and oxidative stress [18,19].

Additionally, different CAT gene family members exhibited obvious functional differentiation. In Arabidopsis, the *atcat2* mutant exhibited a dwarf and disseminated necrotic lesion phenotype, but no obvious phenotypic change was observed in the *atcat1* and *atcat3* mutants compared with the wild type [20,21]. The enzyme activity analysis revealed that the catalase activity in atcat2 knockout plants was only 20% of that of wild-type plants, and the activity in atcat3 was still maintained at 80%, but no obvious change was observed in atcat1 knockout plants [20,22]. Arabidopsis *AtCAT2* plays a leading role in the implementation of CAT enzyme function, which is necessary for scavenging H_2_O_2_ produced by photorespiration [21].

Rice (*Oryza sativa* L.) is one of the most important food crops, with more than half of the world’s population relying on it as a staple food [23], and it is also the model plant for monocotyledonous plant research [24]. It has been reported that rice OsCAT gene expression responded to abiotic stresses and has played a role in disease resistance response [25,26]. However, the CAT gene family in rice genome has not been comprehensively and systematically characterized. Moreover, it is unclear which OsCAT member may play a leading role.

In this study, we first performed a systematic bioinformatics analysis of the OsCAT gene structure, protein localization, promoter elements and phylogenetic tree. Then, the *OsCAT3* gene with the highest expression level among the three OsCAT genes was studied in detail. It was found that the recombinant OsCAT3 protein had H_2_O_2_ scavenging activity. The *OsCAT3_crispr_* transgenic plants were dwarfed and had a low survival rate, and the leaves were small with brown spots. The enzyme activity analysis showed that the CAT, POD and SOD activities were significantly reduced in *OsCAT3_crispr_* transgenic plants. Further microarray analysis indicated that the differentially expressed genes in *OsCAT3_crispr_* transgenic plants were primarily enriched in the toxin metabolism and photosynthesis pathways, and heat stress-related genes were significantly upregulated. Our results lay a solid foundation for understanding the functions and the underlying mechanisms of OsCAT genes, and provide new information for improving rice stress tolerance by genetic engineering.

## 2. Materials and Methods

### 2.1. Plant Materials and Growth Conditions

The rice variety Wu Yun Japonica was used in this study. Rice plants were grown in the Nanchang experimental field of Jiangxi Agricultural University, and materials such as roots, stems, leaves and anthers were collected at different times, immediately frozen in liquid nitrogen and stored at −80 °C.

### 2.2. Bioinformatics Analysis

The CDSs and protein sequences of the three *OsCAT* families in rice (*Oryza sativa* L) were analyzed by homology alignment with the NCBI (www.ncbi.nlm.nih.gov, accessed on 20 August 2022) and reference protein databases using the known protein sequences of the *Arabidopsis* CAT family genes as a reference. The protein sequences were imported into BioXM for protein molecular weight and isoelectric point analysis. A subcellular localization prediction analysis of *OsCAT1*, *OsCAT2*, and *OsCAT3* was performed on the online website WoLF PSORT. Gene structures were mapped using the bioinformatics website GSDS (http://gsds.cbi.pku.edu.cn/, accessed on 25 August 2022) for the full sequences of the CAT genes and CDSs of rice and *Arabidopsis thaliana*. Sequence information on CAT homologues in seven species was obtained by NCBI homology alignment. BoxShade, ClustalX and ClustalX2.0 were applied for simultaneous homology comparison and protein sequence analysis. Evolutionary trees for multiple species were derived using MEGA7 software.

### 2.3. Reverse Transcription–Polymerase Chain Reaction (RT-PCR) and Quantitative Real-Time Polymerase Chain Reaction (qRT−PCR)

Total RNA was extracted using TRIzol reagent according to the instructions for the kit (TaKaRa, Shiga Prefecture, Japan). RNA was reverse-transcribed to cDNA using a reverse transcriptase kit (TaKaRa, Japan). Quantitative real-time PCR (qRT−PCR) was performed using TB Green Fast qPCR Mix (TaKaRa, Japan) and Applied Biosystems 7500. The reaction process was as follows: 5 min at 94 °C, 5 s at 94 °C, 10 s at 55 °C, and 15 s at 72 °C for 40 cycles. Relative expression levels were normalized to actin for quantification by using the 2^−ΔΔCT^ method. Three biological replicates were performed for all the reactions.

### 2.4. Prokaryotic Expression

The CDS of *OsCAT3* and the sequence of the known prokaryotic expression vector pGEX−4T−1 were obtained from the NCBI search, and homologous recombination primers were designed using SnapGene. Rice leaf RNA was extracted using MiniBEST Plant RNA Extraction Kit (TaKaRa, Japan), RNA was reverse-transcribed to cDNA using a reverse transcriptase kit (TaKaRa, Japan). Gel recovery of the target vector pGEX−4T−1 fragment was performed by vector digestion using *BamH* I and *Sal* I; the target fragment was amplified by PCR using homologous recombinant primers and subjected to DNA purification. The In-Fusion HD kit (Takara, Japan) was used to seamlessly construct the prokaryotic expression vector pGEX−4T−1−OsCAT3 and to transform *E. coli*. Single colonies were selected to shake the plasmid, and the positive vector was selected for induction of recombinant protein expression in *E. coli* BL21 after PCR, plasmid double digestion verification and sequencing.

### 2.5. Plant Transformation and the Generation of Transgenic Plants

The *OsCAT3* sequence was downloaded from NCBI, the full length of the resulting sequence was analyzed in the software (http://crispr.mit.edu/, accessed on 7 September 2021) to find the PAM sequence, and the PAM near the promoter region was selected to design the target sequence. The second exon of *OsCAT3*, “CTCGTTCAACGGCCCGCTGTGG”, was selected as the target sequence, and the oligo sequences were designed as follows: sGRNA−CAT3−F−TGTGTGCTCGTTCAACGGCCCGCTG and sGRNA−CAT3−R−AAACCAGCGGGCCGTTGAACGAGCA. The knockout vector was constructed using the CRISPR/Cas vector construction kit and transformed into *E. coli* DH5α to obtain the target vector BGK03−*OsCAT2*. The target vector was then transferred into *Agrobacterium tumefaciens* EHA105 to obtain transgenic plants.

### 2.6. Microarray Analysis of CRISPR/Cas Plants

The control group was cultivar 9522 (*Oryza sativa* ssp. Japonica), and the experimental group was *OsCAT3_crispr_* knockout transgenic plants from the T2 regeneration.

Three replicates of 50 mg each were taken from rice leaves at the seedling stage. Total RNA was extracted from the samples and quantified by spectrophotometer, and the corresponding dilution ratio was chosen according to the starting amount of total RNA. Total RNA from rice leaves was used as a template for in vitro amplification and biotin labelling, followed by in vitro transcription to synthesize cDNA. Lastly, magnetic beads were used to remove salt and enzymes from the synthesized cDNA, and then the cDNA was fragmented to a size suitable for hybridization. After fragmentation, the cDNA was microarrayed, the scan image results are obtained, and the RAM algorithm was applied to normalize and background correct the images to ensure that the data were authentic and reliable. Similarity within sample groups and differences between groups were analyzed before performing differential gene screening, and principal component analysis was used in this experiment. The results of the data analysis by RAM were then screened for differential genes with the R program package, using Q-value and difference ploidy as criteria, with differentially expressed genes being indicated when the Q-value was less than 0.005 and the difference ploidy was greater than 2.

### 2.7. DAB and NBT Staining

Using Servicebio kit plant superoxide anion staining solution (NBT), wild-type and OsCAT3crispr transgenic rice leaves were immersed in NBT working solution, protected from light for 4 h, until the positives appeared dark blue, treated with 95% ethanol for several decolorization times and removed for photography. With Servicebio kit plant peroxide staining solution (DAB), wild-type and OsCAT3crispr transgenic rice leaves were immersed in DAB working solution, protected from the light for 4 h until the positives appeared dark brown, treated with 95% ethanol for multiple decolorization steps and then removed and photographed.

## 3. Results

### 3.1. Bioinformatics Analysis of the CAT Gene Family in Rice

To identify the CAT members in the rice genome, an *Arabidopsis* catalase sequence (GenBank accession number GU248529) was used as a BLAST query sequence to search the related sequences in the database, which then identified the three candidate sequences OsCAT1–OsCAT3 in TIGR pseudomolecule version 5.

**Figure 1:**
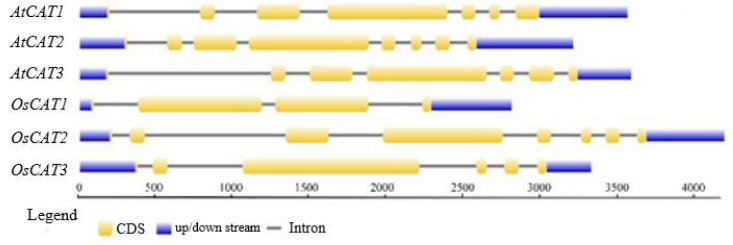
The gene structure of the CAT family genes from *Arabidopsis thaliana* and *Oryza sativa*. Blue color shows the UTR regions, yellow color shows the CDS or exons, black horizontal line shows the introns.

The details on the gene name, locus, chromosome location, protein length, theoretical molecular weight (MW) and isoelectric point (pI) are listed in Table 1. The three *OsCAT* genes were all 1479 bp, and the molecular weight and isoelectric point of the encoded OsCAT protein were basically the same. However, the number of introns varied from three to seven, in addition to having different exon arrangement patterns (Figure 1). The subcellular localization predictions showed that OsCAT1 was more likely to be located in the cytoplasm and plasma membrane, while OsCAT2 and OsCAT3 were more likely to be located in the peroxisome and cell membrane.

To determine the evolutionary relations of CAT genes between rice and other plant species, an unrooted NJ phylogenetic tree using bootstrap analysis (1000 replicates) was constructed based on the protein sequences of *Arabidopsis*, maize, sorghum, millet, soybean and soybean CATs.

**Figure 2:**
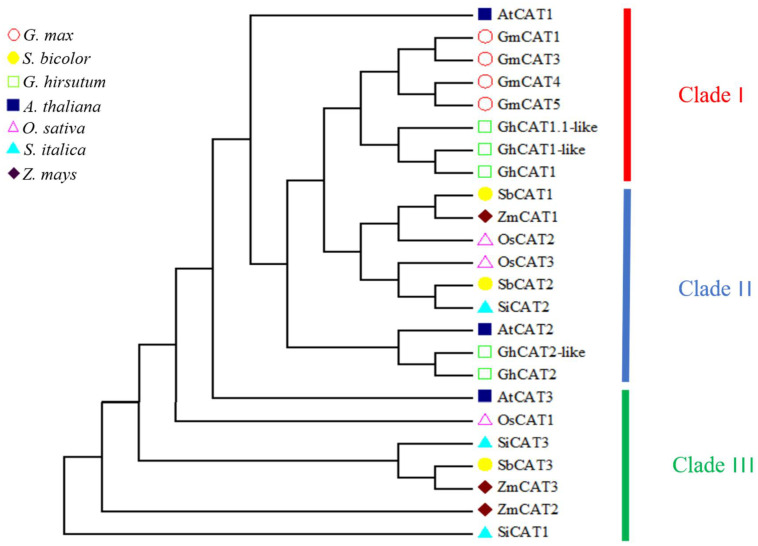
Phylogenetic relationship and architecture of 24 CAT proteins. According to the phylogenetic relationships, the CAT genes from seven species were clustered into three groups (Clade I−III).

As shown in Figure 2, the CAT proteins in various plant species were clearly classified into three classes (I−III) containing eight, nine and seven genes, respectively. OsCAT1 was grouped in class III, and OsCAT2 and OsCAT3 were clustered together in class II. It seemed that OsCAT1 was more closely related to *Arabidopsis* AtCAT3, and OsCAT2 and OsCAT3 were more closely related to *Arabidopsis* AtCAT2. Previous studies have shown that AtCAT2 is essential for the scavenging of H_2_O_2_ produced by photorespiration and plays a leading role in the implementation of CAT enzyme function in *Arabidopsis*. It is speculated that rice OsCAT2 and/or OsCAT3 may have a similar function to that of *Arabidopsis* AtCAT2.

**Figure 3:**
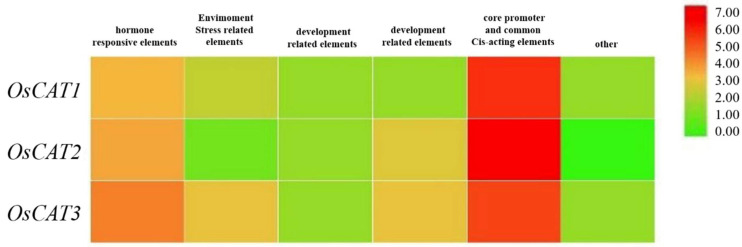
Heat map analysis of upstream elements in the *OsCAT* gene family. In the heat map, the red color shows high enrichment, and the green color shows low enrichment levels.

A cis-element analysis of 2 kb sequences by PlantCARE (http://bioinformatics.psb.ugent.be/webtools/plantcare/html, accessed on 10 September 2022) showed that a total of 12, 14 and 20 specific upstream elements were identified in OsCAT1, OsCAT2 and OsCAT3, respectively (Figure 3). These upstream elements can be divided into six categories: hormone response elements, stress response elements, growth and development-related elements, photoperiod response elements, core promoter and general enhancer elements, and other related elements. The clustering heat map analysis revealed that stress and hormone ring-like response elements showed higher enrichment in the stress response elements of the *OsCAT3* gene. This characteristic may predict the dominance of *OsCAT3* in the scavenging of hydrogen peroxide in rice.

### 3.2. Patiotemporal Expression Patterns of the CAT Gene Family

**Figure 4:**
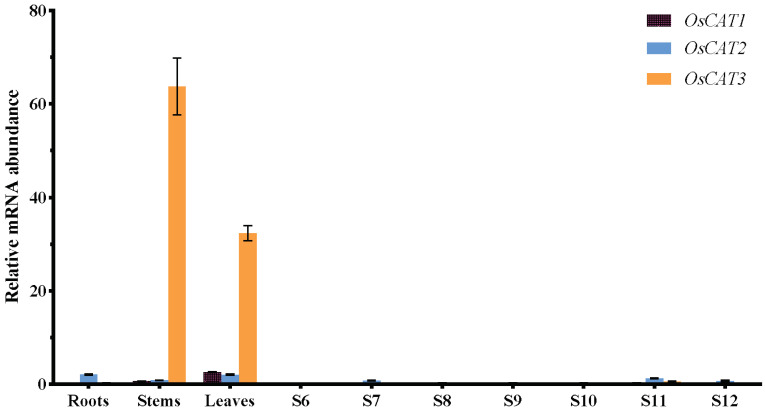
Expression analysis of rice OsCAT1–OsCAT3 in different tissues using qRT−PCR. S6–S12 indicate different periods of anther development. OsACTIN1 was used as the internal standard for each gene. Values indicate the means of three biological replicates ± SE.

To gain insight into the functions of *OsCAT1*–*OsCAT3* in rice, we performed RT−PCR analysis of RNA from roots, stems, leaves and flowers at various stages of development (Figure 4). *OsCAT1* and *OsCAT2* exhibited relatively low expression in all the study tissues. *OsCAT3* showed similarly low expression in flowers and roots but significantly higher expression (a dozen times that of Plant CARE *OsCAT1* and *OsCAT2*) in leaves and stems. Based on the expression level, it was speculated that *OsCAT3* may play a dominant role in rice growth and stress; thereafter, we performed a functional analysis of the *OsCAT3* gene.

### 3.3. OsCAT3 Prokaryotic Expression and Activity Detection

**Figure 5:**
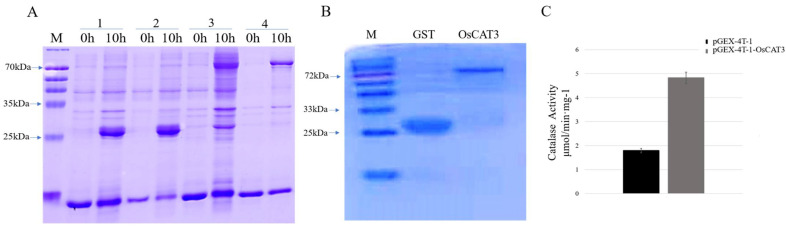
OsCAT3 prokaryotic expression and H_2_O_2_ scavenging activity analysis. (**A**): SDS−PAGE analysis of pGEX−4T−1−OsCAT3 fusion protein in E. coli BL21 strain cells before and after IPTG induction; M. protein maker; 1. gel electrophoresis results of BL/pGEX−4T−1 total protein after 0 and 10 h of induction; 2. gel electrophoresis results of BL/pGEX−4T−1 supernatant proteins after 0 and 10 h of induction; 3. gel electrophoresis results of BL/pGEX−4T−1−OsCAT3 total protein after 0 and 10 h of induction; and 4. gel electrophoresis results of BL/pGEX−4T−1−OsCAT3 supernatant proteins after 0 and 10 h of induction. (**B**): Purification of pGEX−4T−1−OsCAT3 recombinant protein; GST. Gel electrophoresis results of BL/pGEX−4T−1 protein purification; OsCAT3. Gel electrophoresis results of BL/pGEX−4T−1−OsCAT3 protein purification. (**C**): H_2_O_2_ scavenging activity analysis of the purified fusion protein pGEX−4T−1−OsCAT3 in vitro.

OsCAT3 encodes a CAT enzyme that breaks down hydrogen peroxide into water and oxygen by catalyzing the transfer of electrons. To investigate whether the OsCAT3 protein has the ability to scavenge H_2_O_2_, the prokaryotic expression vector pGEX−4T−1−OsCAT3 was constructed for protein expression and in vitro enzyme activity assays. *E. coli* transformants containing the pGEX−4T−1 and pGEX−4T−1−OsCAT3 constructs were induced with 0.2 mM IPTG treatment for 10 h at 18 °C. The total proteins from the transformants following induction were extracted and detected by SDS−PAGE. As shown in Figure 5A, BL/pGEX−4T−1 cells produced a GST protein measuring approximately 26 kDa, while a specific band of approximately 83 kDa was observed in BL/pGEX−4T−1−OsCAT3 cells, which matched the expected size of the band of the OsCAT3 protein plus that of the pGEX−4T−1 protein. It is worth noting that the specific band was observed only in BL/pGEX−4T−1−OsCAT3 cells induced with IPTG, suggesting that the OsCAT3 fusion protein was effectively expressed in *E. coli* cells.

Further analysis showed that the induced OsCAT3 recombinant proteins were observed in both the supernatant and cell pellets of lysed BL/pGEX−4T−1−OsCAT3 cells. Using an affinity chromatography column, the recombinant protein pGEX−4T−1−OsCAT3 in the supernatant was separated and purified successfully (Figure 5B). We then investigated whether the purified pGEX−4T−1−OsCAT3 protein had H_2_O_2_ scavenging capacity using a commercial CAT detection kit. We found that the catalytic capacity of pGEX−4T−1−OsCAT3 was 2.58 of the control pGEX−4T−1, indicating that it has high H_2_O_2_ scavenging activity (Figure 5C).

### 3.4. Analysis of OsCAT3 in CRISPR-Edited Plants

**Figure 6:**
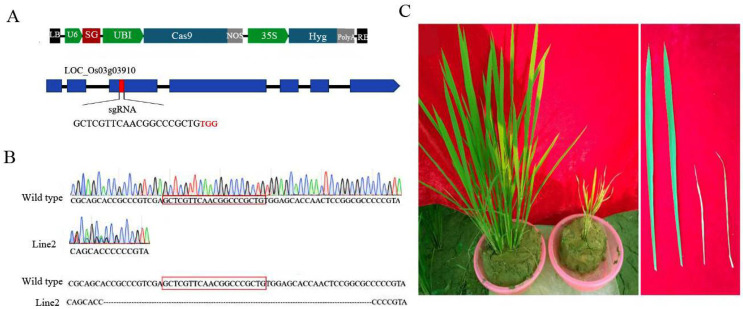
Phenotypes of *OsCAT3_crispr_* transgenic plants. (**A**): Schematic diagram of the *OsCAT3_crispr_* construct. (**B**): Sequence analysis of *OsCAT3_crispr_* transgenic plant. (**C**): Phenotypic analysis of *OsCAT3_crispr_* transgenic plant (**right**) and WT plant (**left**).

To reveal the specific biological functions of OsCAT3 in rice growth and development, the *OsCAT3* gene was edited by using the CRISPR/Cas9 editing technique. First, the 20-bp editing targets (GTGGAGAACCGAACAATAACTGG) in exon 3 were screened as SG sequences using a website (http://crispr.hzau.edu.cn/CRISPR2/, accessed on 7 September 2021), and the targets were cloned into the BGK03 vector to obtain the BGK03−OsCAT3_crispr_ vector (Figure 6A). Subsequently, the BGK03−OsCAT3_crispr_ vector was transferred to rice callus using the *Agrobacterium*-mediated method. A total of 12 independent transgenic positive plants were obtained by regeneration culture and hygromycin resistance screening. Sequencing analysis showed that only line 2 was a homozygous knockout, with a large fragment deletion observed in the target and adjacent areas (Figure 6B). We therefore selected line 2 for subsequent research and named it *OsCAT3_crispr_*. Compared with the wild type, *OsCAT3_crispr_* transgenic plants grew weakly at the seedling stage and had a low survival rate, and the whole plant was dwarfed and yellowish. At the tillering stage, *OsCAT3_crispr_* had fewer tillers, and the leaves were smaller leaves covering brown disease spots (Figure 6C). During the reproductive growth period, the development of the *OsCAT3_crispr_* inflorescence was seriously hindered, and very few seeds were produced.

**Figure 7:**
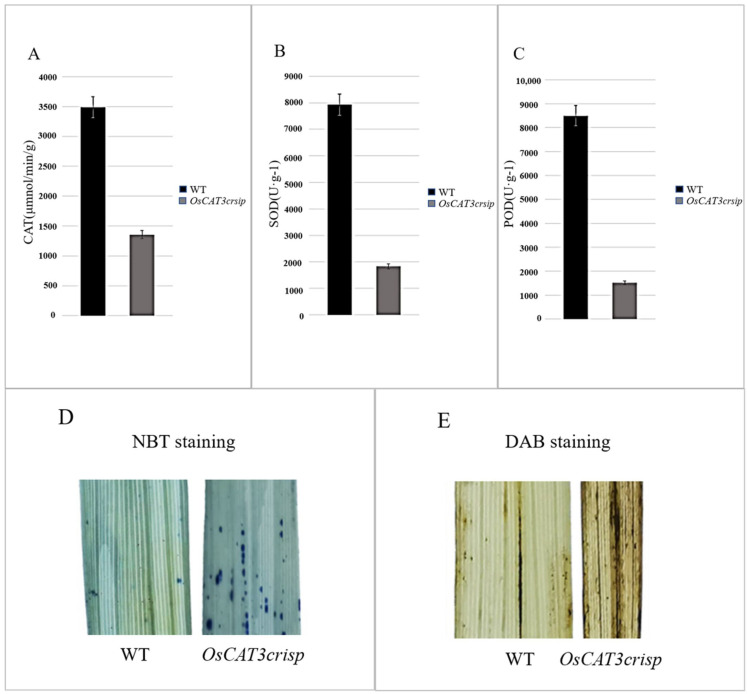
Enzyme activity determination and staining of *OsCAT3_crispr_* transgenic rice. (**A**–**C**): Decreased antioxidant activity of CAT (**A**), SOD (**B**) and POD (**C**) in *OsCAT3_crispr_* plant. Error bars represent SD of three independent experiments. (**D**): NBT staining showing O_2_^−^ accumulation in *OsCAT3_crispr_* leaves. (**E**): DAB staining showing H_2_O_2_ accumulation in *OsCAT3_crispr_* leaves.

The analysis of related physiological enzyme activities indicated that the activities of CAT, POD and SOD were decreased by 57.7%, 75.1% and 69.4%, respectively. Staining assays using NBT and DAB found that stronger blue and brown signals were observed in *OsCAT3_crispr_* transgenic plants, suggesting a higher ROS level in *OsCAT3_crispr_* transgenic plants than in wild-type plants (Figure 7). POD, SOD and CAT are key enzymes that maintain low levels of H_2_O_2_ in cells to prevent damage to normal cellular physiological activities [27].

It was speculated that eliminating OsCAT3 leads to the toxic effect of ROS accumulation on rice and, simultaneously, to a rapid decrease in the activity of POD and SOD, losing their protective effects on cells. The POD and SOD activity decreased rapidly, and the protective effect on cells was lost. The above results revealed that rice OsCAT3 had some antioxidant scavenging ability and played a role in the antioxidant scavenging capacity and stress response in rice. 

### 3.5. Microarray Analysis of OsCAT3_crispr_ Transgenic Plants

To understand the molecular mechanism and regulatory network of OsCAT3, wild-type and *OsCAT3_crispr_* transgenic knockout plants from the T2 generation were used for gene chip analysis. A total of 8812 differentially expressed genes were detected, including 4580 upregulated genes and 4232 downregulated genes.

**Figure 8:**
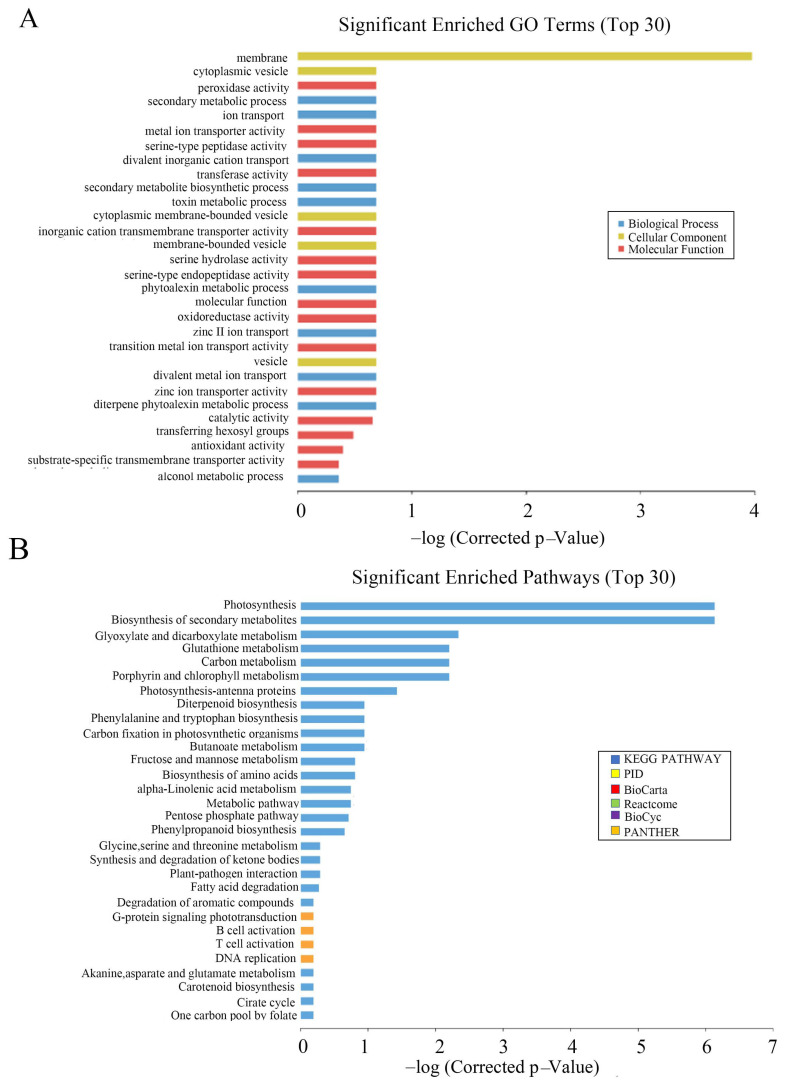
Microarray analysis of *OsCAT3_crispr_* transgenic plants. (**A**): Significant enriched GO Terms of differentially expressed genes. (**B**): Significant enriched pathways of differentially expressed genes.

Through GO functional notes, it was found that differentially expressed genes were significantly enriched in plastids and cell membranes during cell component classification. In molecular function classification, three functional subclasses, molecular function, catalytic activity and transferase activity, and transferring hexosyl groups, were significantly enriched. In the classification of biological processes, a significant enrichment was observed in the diterpene phytoalexin metabolic processes, single-organism biosynthetic process, and phytoalexin biosynthetic process subclasses (Figure 8A).

Interestingly, a series of genes related to antitoxin metabolism were significantly enriched among the differentially expressed genes, including the internal ent-copalyl diphosphate synthase gene (*Os02g0571100*, *OsCPS2*), syn-copalyl diphosphate synthase gene (*Os04g0178300*, *OsCPS4*), cytochrome P450 monooxygenase gene (*Os04g0178400*, *OsCYP99A3*), 9 β−*Pinus densiflora*−7,15−diene synthase gene (*Os04g0179700*, *OsDTS2*) and Neicasa−12,15−diene synthase gene (*Os02g0570400*, *OsDTC1*), two putatively expressed terpenoid synthase genes (*Os12g0491800* and *Os11g0474800*, *OsKSL11*) and one sex-determining gene (*Os04g0179200*, *OsMAS*). Except for *OsCPS1*, which showed 11.25−fold downregulation, the other eight genes were upregulated between 2.42− and 37.51−fold (Table S1).

To explore the changes in metabolic pathways in *OsCAT3_crispr_* transgenic rice, Kyoto Encyclopedia of Genes and Genomes (KEGG) was used to analyze its metabolic regulation pathway. It was found that photosynthesis, biosynthesis of secondary metabolites and glyoxylate and dicarboxylate metabolism were more enriched in the top 30 pathways (Figure 8B). Most genes in the metabolic pathway related to rice photosynthesis were significantly downregulated in *OsCAT3_crispr_* transgenic knockout plants. The most downregulated gene was NADPH (FC = −28.9), which encodes protochlorophyllide oxidoreductase A. Other genes included the chlorophyll A–B binding protein gene (FC = −25.4), the magnesium protoporphyrin O-methyltransferase gene (FC = −17.9) and the photosystem II 11 kD protein gene (FC = −12.9).

## 4. Discussion

### 4.1. OsCAT3 May Play a Dominant Role in the Implementation of Rice CAT Function

Catalase (CAT) is an important antioxidant enzyme in organisms that is generally present in the form of a multigene family. For example, there are three CAT genes in *Arabidopsis*, tobacco, corn, pumpkin and cucumber, seven CAT genes in cotton and fourteen CAT genes in rape [9,10,11,12,13,14,28,29]. Bioinformatic analysis revealed that three rice OsCATs had distinct differences in gene structure and subcellular localization. The analysis of the evolutionary tree indicated that CAT genes in different species can be divided into three groups. OsCAT3 was grouped into a Clade II subclade and was more closely related to Arabidopsis AtCAT2. It was reported that AtCAT2 played a dominant role in CAT enzyme function in Arabidopsis. The upstream sequence analysis showed that cis-elements associated with hormone-responsive elements and stress-responsive elements were significantly enriched in the *OsCAT3* promoter region.

Based on the bioinformatics analysis results, it was speculated that *OsCAT3* may be the most important OsCAT gene in rice. Then, we also studied *OsCAT3* function through relevant experiments. It was found that OsCAT3 prokaryotic expression protein has an obvious ability to remove H_2_O_2_, and the enzyme activity was 2.58 times that of the control. Additionally, the *OsCAT3_crispr_* knockout transgenic line grew dwarfed plants with albino leaves, and the activities of CAT, SOD and POD antioxidant enzymes decreased significantly. The rice *OsCAT3_crispr_* knockout plant exhibited a similar phenotype to that of Arabidopsis *AtCAT2*. Combined with the bioinformatics analysis results, we believe that *OsCAT3* may play a dominant role in the implementation of rice OsCAT function.

OsCAT1 belongs to the Clade III subfamily. Previous studies have shown that Clade III is an unnecessary subfamily, and its specific functions require further study.

OsCAT2 and OsCAT3 are evolutionarily assigned to the Clade II subfamily, and both are predicted to be localized in cell membranes and peroxisomes, but they show distinct differences in expression patterns. Therefore, we hypothesize that OsCAT2 shows significant functional differentiation and may regulate other rice developmental processes. It is speculated that OsCAT2 showed functional differentiation and may regulate other rice development processes.

In fact, the involvement of CAT genes in plant growth and development has been reported in other species. In potatoes, the overexpression of catalase and glycolate oxidase genes could increase the plant height, leaf area and effective leaf number. Additionally, the potato weight increased, but some plants showed tuber deformities [30]. In Scots pine, CAT is involved in embryogenesis and cell death processes [31]. In cotton, *GhCAT1*−*GhCAT4* were highly expressed throughout all the developmental stages, implying an extensive function during cotton growth and development [29]. Of course, these members may also serve both to scavenge ROS and to regulate plant growth and development. 

*OsCAT3* is the most dominant CAT gene, and it can be used to study the detailed mechanism of action and be further applied in production practice. It can also provide theoretical clues for related studies in other species.

Because *OsCAT3* may be the most dominant OsCAT gene, we can use the *OsCAT3_crispr_* knockout mutant to deeply study its molecular mechanism and further apply it to production practice in the future [4].

### 4.2. Possible Molecular Mechanism of OsCAT3

Among the differentially expressed genes, the most significantly downregulated genes were associated with the formation of chloroplasts or chloroplast-like vesicles. Further enrichment analysis of KEGG pathways showed that differentially expressed genes related to the photosynthesis pathway were obviously downregulated.

The most downregulated gene, *SPP* (*Os06g0625400,* downregulated 83.33 times), was involved in the formation of chloroplast precursors in the cell structure, and another gene, *NYC1* (*Os05g0462000*, downregulated 80.00 times), was primarily related to the degradation of light capture complex II and chloroplast grana during leaf senescence [32,33].

It was speculated that the mutation of the *OsCAT3* gene significantly affects rice photosynthesis and development processes, leading the *OsCAT3_crispr_* knockout plants to show dry leaves covered with white spots and diseased spots.

Among the differentially expressed genes, the gene with the most obvious upregulated change was related to heat shock protein (HSP). The largest upregulated (1122.57 times) gene was *OsHSP26.7* (*Os03g0245800*). HSP is a stress protein produced by organisms in adverse environments, and it is related to stress resistance [34].

The current research showed that when plants are exposed to heat shock or other environmental stresses, they will cause structural denaturation and functional disorder of proteins. Overexpression of *HSP* genes can maintain the configuration of proteins, thus regulating the dynamic balance of cells [34]. It is hypothesized that there may be negative feedback regulation between HSP and *OsCAT3* expression, and knocking out *OsCAT3* leads to the destruction of its stress resistance function. To adapt to the environment for growth and development, plants need to increase their expression of HSP genes to maintain normal cell metabolism. The analysis of biological processes revealed that several metabolic processes involved in antitoxins were significantly enriched in the differentially expressed genes, and nine toxin genes were significantly upregulated.

Phytoalexins are a group of low molecular weight substances that are synthesized and then accumulate in plants when they are attacked by biotic or abiotic factors, which protect them against disease infection. In potatoes, exogenous treatment with H_2_O_2_ could induce the production of plant antitoxins [35]. It is hypothesized that *OsCAT3* knockdown leads to the accumulation of hydrogen peroxide and induces the synthesis of plant antitoxins. The upregulated genes related to plant antitoxin may have resulted from the accumulation of hydrogen peroxide in *OsCAT3_crispr_* knockout plants.

In summary, we performed a comprehensive characterization of the OsCATs in rice through analysis of their gene structures, promoter sequences, phylogenetic relationships and the expression patterns, suggesting the *OsCAT3* maybe the most important member. Further study indicated OsCAT3 prokaryotic expression protein has an obvious ability to remove H_2_O_2_, and the *OsCAT3_crispr_* plant showed severely stunted growth with reduced antioxidant enzymes activities. Combined with the bioinformatics analysis results, we believe that OsCAT3 may play a dominant role in the implementation of rice OsCAT function. Our results provide important information for further functional research on OsCAT gene family in rice.

## Figures and Tables

**Table 1:** CAT gene and coding protein information.

Gene ID	Gene Name	Chr	CDS Length (bp)	Protein (aa)	MW (kDa)	Theoretical pI	Predicted Location
*Os02g02400*	*OsCAT1*	2	1479	492	56.70	7.0063	Cyto and Memb
*Os06g51150*	*OsCAT2*	6	1479	492	56.59	6.9562	Pero and Memb
*Os03g03910*	*OsCAT3*	3	1479	492	56.77	7.4500	Pero and Memb

## Data Availability

All data generated or analyzed during this study are included in this published article and its Supplementary Information Files.

## Supplementary Materials

The following supporting information can be downloaded at: https://www.mdpi.com/article/10.3390/genes14010138/s1; Table S1: The differentially expressed genes associated with toxin metabolism of *OsCAT3_crispr_*.
