# Supplementary material for: Analysis of CAT Gene Family and Functional Identification of OsCAT3 in Rice
Source: Genes (Basel). 2023 Jan 4;14(1):138. doi: 10.3390/genes14010138 (PMC9858675; doi:10.3390/genes14010138)
Supplement: Supplementary file 1 [file genes-14-00138-s001.zip › genes-2099850-supplementary.pdf]

Table S1. The differentially expressed genes associated with toxin metabolism of *OsCAT3*$_{crispr}$

Gene ID	Name	FC
Os02g0571100	*OsCPS2; OsCyc2*	+2.42
Os04g0178300	*OsCPS4; OsCyc1*	+15. 24
Os02g0278700	*OsCPS1*	-11.25
Os12g0491800	terpene synthase	+20.51
Os04g0178400	*CYP99A3*	+6.03
Os04g0179700	*OsDTS2; OsKSL4; KS4*	+37.51
Os02g0570400	*OsDTC1; OsKS3*	+25.92
Os04g0179200	*OsMAS*	+10.43
Os11g0474800	*OsKSL11*	+7.05
